# Evidence that Illness-Compatible Cues Are Rewarding in Women Recovered from Anorexia Nervosa: A Study of the Effects of Dopamine Depletion on Eye-Blink Startle Responses

**DOI:** 10.1371/journal.pone.0165104

**Published:** 2016-10-20

**Authors:** Caitlin B. O’Hara, Alexandra Keyes, Bethany Renwick, Katrin E. Giel, Iain C. Campbell, Ulrike Schmidt

**Affiliations:** 1 King’s College London, Institute of Psychiatry, Psychology & Neuroscience, Department of Psychological Medicine, Section of Eating Disorders, London, United Kingdom; 2 Medical University Hospital Tübingen, Department of Psychosomatic Medicine and Psychotherapy, Tübingen, Germany; Erasmus University Rotterdam, NETHERLANDS

## Abstract

In anorexia nervosa (AN), motivational salience is attributed to illness-compatible cues (e.g., underweight and active female bodies) and this is hypothesised to involve dopaminergic reward circuitry. We investigated the effects of reducing dopamine (DA) transmission on the motivational processing of AN-compatible cues in women recovered from AN (AN REC, n = 17) and healthy controls (HC, n = 15). This involved the acute phenylalanine and tyrosine depletion (APTD) procedure and a startle eye-blink modulation (SEM) task. In a balanced amino acid state, AN REC showed an increased appetitive response (decreased startle potentiation) to illness-compatible cues (underweight and active female body pictures (relative to neutral and non-active cues, respectively)). The HC had an aversive response (increased startle potentiation) to the same illness-compatible stimuli (relative to neutral cues). Importantly, these effects, which may be taken to resemble symptoms observed in the acute stage of illness and healthy behaviour respectively, were not present when DA was depleted. Thus, AN REC implicitly appraised underweight and exercise cues as more rewarding than did HC and the process may, in part, be DA-dependent. It is proposed that the positive motivational salience attributed to cues of emaciation and physical activity is, in part, mediated by dopaminergic reward processes and this contributes to illness pathology. These observations are consistent with the proposal that, in AN, aberrant reward-based learning contributes to the development of habituation of AN-compatible behaviours.

## Introduction

Anorexia nervosa (AN) involves extreme dietary restriction and aberrant thoughts related to food and weight and it has been proposed that symptoms are related to a diminished ability to experience “reward” (*i*.*e*., positive reinforcement) to natural reinforcers (physical anhedonia) [1, 2]. This proposal may however be too broad as people with AN do experience “reward” (*i*.*e*., positive reinforcement). For example, while they have an aversion or disliking towards energy-dense foods [3], they are preoccupied with eating [2], show increased motivation towards low-calorie foods [4] and exercise [5, 6], and often resist treatment due to the egosyntonic or rewarding nature of symptoms [7]. Importantly however, AN is a disorder of complex aetiology. Therefore, the extent to which altered reward processes are overlapping with genetic, neurobiological, and psychosocial factors [7], or with the physiological consequences of starvation [8], in AN is unknown. Nevertheless, several recent aetiological models propose that AN is, in part, due to a learning of illness-compatible behaviours which have become habitual and therefore difficult to change [9–11].

In relation to food, functional brain imaging (fMRI) studies of people with AN show both increased and decreased activation in regions involved in evaluating rewarding and aversive food stimuli [12–20]. However, similar investigations on responses to thinness and exercise cues, although understudied, are relatively consistent. In response to underweight (relative to normal weight) stimuli, both adolescents [21] and adults [22] with AN show increased activation in the ventrostriatal dopaminergic reward system, whereas healthy controls (HC) show the opposite. In response to stimuli depicting physical activity, women with AN (relative to athlete and non-athlete controls) show heightened activation of the prefrontal cortex, suggesting that increased inhibitory resources are required to maintain behavioural performance during exposure to exercise cues in this group. Physical activity related stimuli could lead to an augmented prefrontal inhibitory response due to biases elicited by the illness-specific information, in that exercise cues are perceived as more rewarding in individuals with AN [23]. Together, these data suggest that illness is associated with an increased drive towards cues consistent with illness-specific goals (*e*.*g*., thinness and engaging in exercise) and challenge a broad “anhedonia” hypothesis of AN [1]. It is possible that increased neural activity in reward-related circuits in response to illness-specific stimuli may, over time, heighten their incentive salience, and this may promote/maintain behaviours associated with AN [9]. This is supported by disease models that implicate corticostriatal systems in its aetiology [2, 10–12] or, more specifically, a heightened motivational drive (or ‘wanting’) towards AN-compatible cues (*e*.*g*., thinness, exercise) in the perpetuation of its core symptoms [9].

‘Wanting’ is a process of incentive salience attribution which mainly involves mesolimbic dopaminergic reward processes, and alterations in these have been implicated in the development and maintenance of AN symptomatology [9, 24, 25]. For example, fMRI studies have reported that individuals with AN show increased responses to underweight cues in the ventral striatum, a dopamine (DA) rich brain region implicated in reward [22]. Moreover, people recovered from AN are reported to have increased DA D2/D3 receptor binding potential in the anterior-ventral striatum, suggesting altered DA function, or increased receptor density or affinity [13]. These studies associating DA function with AN are supported by animal data reporting that chronic food restriction sensitizes the mesolimbic DA system [26]. Rodent models of AN also implicate dopaminergic reward processes in that susceptible rats express increased DA D2 receptor levels in the caudate-putamen [27] and show reduced activity and weight loss, and increased food intake in response to DA antagonists [28, 29]. These data suggest that heightened sensitivity in dopaminergic circuits is related to illness pathology [25].

Psychophysiological methods (*e*.*g*., eye-tracking, visual probe and electromyography) provide a means of exploring implicit biases to motivationally salient cues. Eye-tracking studies have shown that gaze is preferentially allocated towards rewarding cues, suggesting that attention orientation and engagement are indicative of an appetitive response [30]. In a study of individuals with AN and HC, no group differences were observed in early attentional allocation towards high-calorie food images, but participants with AN showed heightened later attentional avoidance of such cues [30]. Similar methods indicate that patients with AN and athletes (relative to HC) show heightened attentional engagement toward active versus non-active images [31]. Eye-tracking and visual probe methods have also identified attentional biases towards negative appearance-related stimuli (i.e., larger female body pictures) and negative self-referential body images (i.e., own ‘ugly’ body parts) in individuals with AN relative to controls [32–34].

Facial electromyographic (EMG) methods (*e*.*g*., startle eye-blink modulation (SEM), and zygomatic/corrugator muscle reactivity analysis) are sensitive to automatic motivational states of approach (appetitive) and withdrawal (aversive). Exposure to aversive motivational states increases the intensity of the startle eye-blink and corrugator muscle amplitudes, whereas appetitive motivational states decrease the intensity of the startle eye-blink reflex while increasing zygomatic muscle reactivity [35, 36]. Studies using these techniques have provided little evidence that pictorial food cues are automatically evaluated as unpleasant in individuals with AN compared to HC [37, 38]. Similarly, SEM analysis of responses to images of healthy slim females found no motivational biases in women with AN relative to HC [37]. However, both self-report ratings and functional brain imaging patterns indicate that individuals with AN attribute heightened salience to underweight relative to healthy weight bodies [21, 22]. Thus, it may be necessary to extend these paradigms to AN-compatible stimuli (*e*.*g*., underweight cues) to elucidate the appetitive and aversive processes related to illness.

Individuals with acute AN have widespread alterations in central- and peripheral-organ systems. This is a major research confound in that it becomes difficult to determine whether changes are a cause or consequence of starvation. Importantly, however, women recovered from AN often show incomplete normalization of illness-related behaviours as well as altered neural reward processing of food cues [15, 16, 18, 20], suggesting that these differences reflect premorbid traits that contribute to illness vulnerability. Alternatively, observed differences may reflect scarring effects of illness. To avoid the confounding effects of malnutrition and to investigate state- vs trait-aspects of illness, we recruited women recovered from AN (AN REC) and HC. We used a SEM task with acute phenylalanine/tyrosine depletion (APTD) to investigate whether lowering DA influences motivational processing of AN-compatible cues.

The study is underpinned by two hypotheses. The AN REC group (relative to HC) will show an appetitive response (decreased startle potentiation) towards stimuli consistent with egosyntonic symptomatology (*i*.*e*., underweight bodies and physically active bodies). Secondly, in AN REC (relative to HC), lowering DA will decrease motivation towards such cues as reflected by no change in startle potentiation to illness compatible cues (relative to neutral stimuli).

## Materials and Methods

### Participants

Nineteen adult women recovered from AN were recruited via Beat, the UK’s eating disorder (ED) charity, and using a King’s College London circular e-mail. Recovery was defined as: maintaining weight >85% of average body weight, not having binged, purged, or engaged in significant restrictive eating patterns and/or other compensatory behaviours, all for at least 1 year before the study, and no clinically significant scores (≥2.80) on the Eating Disorders Examination Questionnaire (EDE-Q) [39]. Seventeen HC women were recruited using a University circular e-mail. Controls were matched for age and BMI, and reported no history of an ED or any other psychiatric illness.

Exclusion criteria were: insufficient knowledge of English, a significant medical illness (*e*.*g*., a cardiovascular or neurological disorder), substance abuse/dependence (including smoking >10 cigarettes/day), presence of an Axis I psychiatric disorder needing treatment in its own right, and pregnancy. AN REC participants who were taking selective serotonin reuptake inhibitors (SSRIs) were not excluded, provided they had been on a stable dose for at least 3 months. Individuals were excluded if they were on any form of psychotropic medication that targeted DA more directly (e.g., antipsychotics). The study was approved by the London/West London Research Ethics Committee (ref 11/LO/1082). Informed written consent was obtained from all volunteers. Participants were compensated with £100.

### Procedure

Two sessions were scheduled a minimum of three days apart. On the day prior to testing, participants followed a low protein diet and were asked to fast and abstain from caffeine and/or smoking from midnight. Consumption of alcohol was forbidden in the 24h preceding testing. On the morning of each session, height and weight were measured, and baseline ED pathology and reasons for exercise were assessed using the EDE-Q [39] and the Reasons for Exercise Inventory (REI) [40], respectively. Pre- and post-session, the Depression, Anxiety, and Stress Scales (DASS-21) [41] were used to measure changes in mood. Blood (5ml) was used for analysis of baseline plasma amino acids (AA).

Participants then ingested one of two AA mixtures, one deficient in DA’s precursors, phenylalanine and tyrosine (APTD), or one nutritionally balanced control mixture (BAL). In the APTD method, participants ingest a mixture of essential amino acids (AA) that is deficient in DA’s precursors, phenylalanine and tyrosine. Ingestion of the AA load induces protein synthesis, diminishing the body’s stores of phenylalanine and tyrosine [42]. Furthermore, the APTD mixture contains other large neutral amino acids (LNAAs), and thus competitive inhibition of the catecholamine precursors across the blood-brain barrier is also increased [43], further reducing the amount of phenylalanine and tyrosine in the brain [44, 45]. The behavioural effects of APTD are contrasted with those observed in the BAL control condition. The BAL mixture contains phenylalanine and tyrosine, and thus, similar to all other LNAAs, increases in their concentrations are observed. Composition and preparation of these were based on the full 100g mixture used previously [43, 46], with each component reduced by ~20% to account for lower weight in women [43] (S1 Appendix). Participants were allocated at random to BAL or APTD first in accordance with a counterbalanced cross-over design to control for order effects. Volunteers and researchers were blind to treatment allocation. Manufacturing and randomization was conducted by the Royal Victoria Infirmary pharmacy (Newcastle-Upon-Tyne).

Four hours after ingestion of the AA mixture, a second blood sample was drawn. This was followed by the SEM task, which was based on previous methods used in eating disorders [37]. Participants, wearing headphones, were seated at a screen and startle responses were elicited using a 50ms 104-dB broadband noise probe over a 70dB continuous broadband background noise. The startle eye-blink reflex was detected using three miniature silver/silver chloride electromyography electrodes: two recording electrodes were attached to the musculus orbicularis oculi beneath the left eye, and one reference electrode was attached behind the left ear. After an acclimatization period (5min), participants were presented with 90 pictorial stimuli depicting ED-relevant and neutral cues (18 images / stimulus category). Images were separated into three equivalent blocks and were shown in a fixed random order. Each picture was presented for 6s, followed by a varying inter-trial interval (ITI) of 3-9s of a blank screen. Twelve of the 18 pictures in each category were accompanied by an acoustic probe presented between 2 and 4.5s after picture onset. Additionally, 12 startle probes were presented between two ITIs as a blank slide to enhance unpredictability. After the task, subjective ratings for startle stimuli were measured using 10cm visual analogue scales (VAS).

Tyrosine and phenylalanine plasma levels were measured by high-performance liquid chromatography and fluorometric detection (HyPURITY, Thermo Electron Corporation). Samples were missing from 4 AN REC and 3 HC participants.

### Pictorial Stimuli

Healthy body pictures depicted images of slim female models dressed in clothing which highlighted their shape [37]. To reduce group differences in subjective processing of facial emotions, no head or face was visible. Images of underweight bodies were collected by a systematic web-image search of pro-anorexia blogs. They were chosen to match the healthy body images on colour, complexity, visible body parts, and valence. Exercise stimuli were coloured photographs of a female athlete engaging in physically active or physically passive motions. These had been prepared for a study on attentional processing of hyperactivity information in AN and were pre-tested for valence, arousal, and estimation of physical strain [31]. Neutral stimuli depicting household furniture items were used previously and matched for colour, complexity, valence, and arousal [47].

### Equipment and Data Reduction

A startle eye-blink monitoring system (SRH-Lab, San Diego Instruments) produced the acoustic probes and recorded eye-blink responses. Raw EMG waveforms (millivolts) were filtered through a 60 Hz notch filter, smoothed by a rolling average of 10 successive points, and scored for peak startle amplitude, peak onset latency (*i*.*e*., number of milliseconds until peak response), and response probability (*i*.*e*., % of valid startle responses). Peak startle amplitude was corrected for baseline (the average EMG activity during the 20ms pre-stimulus period) and was acquired within 21 and 150ms following stimulus onset. Trials were excluded from analyses for the following reasons: no clear peak amplitude detected; unstable baseline recording; or failure to reach peak amplitude within 95ms of onset latency. These trials were used to calculate the response probability. Participants demonstrating a lack of startle response were excluded from the analyses. Response probabilities for each picture category were above 85%. EMG recording and analysis was performed in accord with standard guidelines for human startle EMG research [48].

### Sample Size Calculation

An *a-priori* power calculation based on repeated measures ANOVA (F-test, within-between interaction) determined that a total sample size of 26 (*i*.*e*., 13 participants per group) would have 95% power to detect a medium effect size of 0.30 with a 0.05 two-sided significance level. Adding a drop-out correction factor (1/1-a) with attrition a = 0.10 per group and based on previous behavioural APTD studies [49, 50], we aimed to recruit a total sample size of 32 (*i*.*e*., 16 participants per group).

### Statistical Analyses

Statistical analyses used SPSS Statistics, version 21.0. An alpha level of 0.05 was used for all tests, which were two-tailed. Logarithmic transformations and/or robust bootstrapping equation methods based on 1000 bootstrap samples were used when assumptions of normality and homogeneity of variances were violated.

Independent samples t-tests were used to compare group differences in baseline characteristics. A repeated measures ANOVA with group (HC, AN REC) as the between-subjects factor, and drink (BAL, APTD) and picture category (ED-related stimuli, neutral stimuli) as the within-subjects factors, was conducted to investigate within- and between-group differences in startle response in the two AA conditions. Subjective picture ratings for each picture category were analysed using similar repeated measures ANOVA methods with group as the between-subjects factor, and drink and picture category as the within-subjects factors. To control for possible relationships between appetitive/aversive drive towards AN-specific cues and differences in eating pathology, reasons for exercise, and mood, self-report data assessing these variables were correlated (Pearson, two-tailed) with startle data. Startle difference scores (subtracting startle amplitudes for neutral cues from those for AN-specific cues) were used for correlation analyses. All *post-hoc* t-tests were corrected for multiple comparisons using Bonferroni corrections. Means ± SD are reported; Cohen’s d and partial eta squared (*η*^2^) effect sizes are reported for independent samples t-tests and ANOVAs, respectively.

## Results

### Participants

Of the women recruited (19 AN REC, 17 HC), 17 AN REC and 15 HC completed the study: three (2 AN REC, 1 HC) withdrew due to inability to ingest, or sickness, following the amino acid (AA) drink, and the fourth (HC) relocated. In accordance with startle modulation guidelines, 2 AN REC participants and 1 HC were excluded from analyses due to lack of startle response. Startle data were therefore recorded from 15 AN REC and 14 HC. Data from individuals with BN suggest that SSRIs might impact startle patterns to ED-related cues [37]. *A priori* exploratory analysis showed no group differences between AN REC taking SSRI medication (n = 7) and those who were not (n = 8), thus analyses apply to the whole AN REC group (S1 Table). Importantly however, visual inspection of the data suggests that group effects may be driven by lower startle potentiation to AN-compatible cues among individuals recovered from AN who were not taking medication. This is consistent with previous data showing that SSRI medication in individuals with BN suppresses startle attenuation in response to illness-compatible cues [37]. Nevertheless, the current sample size is insufficient to examine startle eyeblink differences between the SSRI medication subgroups.

No significant group differences with respect to age, ethnicity, education, BMI, or exercise (hours/week) were observed. However, the AN REC (relative to HC) had higher eating and mood disorder symptoms; although no participant scored within the clinical range. AN REC also rated “improving tone” as a more important reason to exercise compared to HC (Table 1).

**Table 1:** Participant Baseline Characteristics.

Baseline Characteristics	AN REC (n = 17)	HC (n = 15)	Statistics: AN REC to HC
Age (years)	24.65 ± 5.24	23.14 ± 3.18	t (30) = -0.97, p = 0.34, ES = 0.35
Ethnicity	White: 13; Black: 0; Hispanic: 1; Other: 3	White: 11; Black: 1; Hispanic: 1; Other: 2	χ^2^(3) = 1.25, p = 0.74
Education (years)	17.82 ± 2.92	17.20 ± 2.08	t (30) = -0.69, p = 0.50, ES = 0.25
BMI (kg/m^2)^	21.45 ± 2.13	21.74 ± 1.58	t (30) = 0.57, p = 0.67, ES = 0.21
Exercise (hours/week)	3.69 ± 2.52	2.64 ± 1.46	t (28) = -1.36, p = 0.18, ES = 0.50
EDE-Q, Global Score**	1.55 ± 1.01	0.38 ± 0.34	t (30) = -4.45, p < 0.01, ES = 1.63
DASS-21, Total Score**	26.65 ± 16.43	9.87 ± 10.18	t (30) = -3.42, p < 0.01, ES = 1.25
REI, Weight Control	4.59 ± 1.50	3.63 ± 1.49	t (30) = -1.82, p = 0.08, ES = 0.67
REI, Attractiveness	3.57 ± 1.42	3.88 ± 1.42	t (30) = 0.61, p = 0.55, ES = 0.22
REI, Tone**	4.55 ± 1.58	2.73 ± 1.57	t (30) = -3.25, p < 0.01, ES = 1.19
REI, Health	4.57 ± 1.18	5.16 ± 1.50	t (30) = 1.22, p = 0.23, ES = 0.45
REI, Fitness	5.13 ± 1.13	4.93 ± 1.18	t (30) = -0.49, p = 0.63, ES = 0.18
REI, Mood	4.60 ± 1.21	4.00 ± 1.65	t (30) = -1.17, p = 0.25, ES = 0.43
REI, Enjoyment	2.49 ± 1.04	2.76 ± 1.80	t (30) = 0.53, p = 0.60, ES = 0.19

*Legend*: AN REC: anorexia nervosa recovered. BMI: body mass index. DASS: Depression, Anxiety, and Stress Scales. EDE-Q: Eating Disorders Examination Questionnaire. ES: Cohen’s d effect size. HC: healthy controls. REI: Reasons for Exercise Inventory. SD: standard deviation. Data are expressed as Means ± SD.

** *P ≤ 0.01*.

On the APTD test session, plasma concentrations of tyrosine and phenylalanine decreased significantly, as reflected by significant Drink by Time interactions (Phenylalanine F(1) = 149.07, p < 0.01, η2 = 0.87; Tyrosine F(1) = 242.03, p < 0.01, η2 = 0.91). It decreased phenylalanine and tyrosine levels by 80.93% and 73.60%, respectively, in HC, and by 78.43% and 73.14%, in AN REC. In contrast, the BAL mixture increased phenylalanine and tyrosine levels by 242.15% and 248.15% in HC, and by 293.33% and 257.30%, in AN REC. Ingestion of both mixtures also significantly decreased the ratios of plasma phenylalanine and tyrosine to other LNAA (p < 0.01). However, drink x time interactions revealed that these reductions were significantly more pronounced in the APTD relative to the BAL condition: tyrosine (AN REC: % 93.33 vs % 46.17, HC: % 96.11 vs % 41.67, F = 9.07, p < 0.01); phenylalanine (AN REC % 94.62 vs % 35.71, HC: % 97.00 vs % 46.15), F = 8.66, p < 0.01) (Table 2).

**Table 2:** Plasma phenylalanine (PHE) and tyrosine (TYR) concentrations (μmol/l), and ratios of tyrosine and phenylalanine to large neutral amino acids (LNAA) at baseline (am) and 4 hour following amino acid ingestion (pm).

Plasma Amino Acids	AN REC (n = 13)						HC (n = 12)					
	BAL (am)	BAL (pm)	APTD (am)	APTD (pm)	%BAL	%APTD	BAL (am)	BAL (pm)	APTD (am)	APTD (pm)	%BAL	%APTD
PHE	53.69 ± 6.63	**162.62 ± 55.44	51.69 ± 5.19	**11.15 ± 5.44	242.15	- 78.43	53.17 ± 5.31	**128.75 ± 50.45	53.33 ± 5.65	**10.17 ± 4.02	293.33	- 80.93
TYR	49.39 ± 10.79	**127.08 ± 26.12	48.69 ± 7.98	**13.08 ± 3.09	248.15	- 73.14	47.25 ± 7.03	**117.25 ± 38.03	50.83 ± 9.50	**13.42 ± 3.58	257.30	- 73.60
PHE:LNAA	0.14 ± 0.01	**0.09 ± 0.03	0.13 ± 0.02	**0.007 ± 0.005	- 35.71	- 94.62	0.13 ± 0.02	**0.07 ± 0.03	0.20 ± 0.25	**0.006 ± 0.003	- 46.15	- 97.00
TYR:LNAA	0.13 ± 0.02	**0.07 ± 0.04	0.12 ± 0.02	**0.008 ± 0.004	- 46.15	- 93.33	0.12 ± 0.02	**0.07 ± 0.05	0.18 ± 0.21	**0.007 ± 0.003	- 41.67	- 96.11

*Legend*: Plasma PHE and TYR concentrations observed at baseline (am) and 4 hours post amino acid drink consumption (pm). Results are reported for both the balanced (BAL) and the acute phenylalanine/tyrosine depletion (APTD) conditions. Data are expressed as Means ± SD. APTD resulted in a significant lowering of plasma PHE and TYR concentrations 4 hours post amino acid drink consumption.

** *P < 0*.*01*. AN REC: anorexia nervosa recovered. HC: healthy controls. SD: standard deviation. %: % difference.

### Startle Responses to ED-Compatible Stimuli

**Fig 1:**
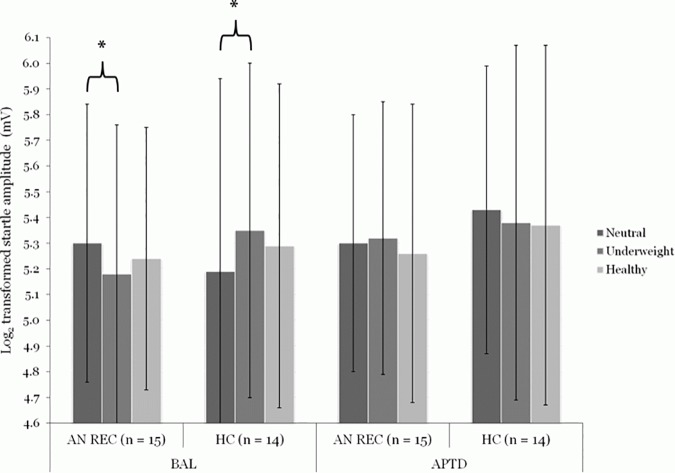
Effect of acute phenylalanine and tyrosine depletion (APTD) on startle eye-blink amplitudes to underweight and healthy female body stimuli (relative to neutral cues) in individuals recovered from anorexia nervosa (AN REC, n = 15) and healthy controls (HC, n = 14). In the balanced condition (BAL), AN REC showed decreased startle potentiation (an appetitive response) to underweight (5.18 ± 0.58) relative to neutral (5.30 ± 0.54) cues, while HC displayed increased startle potentiation (an aversive response) to underweight (5.35 ± 0.65) relative to neutral (5.19 ± 0.75) cues. In the low DA condition (APTD), AN REC perceived underweight stimuli (5.32 ± 0.53) as more aversive than neutral cues (5.30 ± 0.50), while HC perceived neutral stimuli (5.43 ± 0.56) as more aversive than underweight cues (5.38 ± 0.69); however, during APTD, the differences were not significant. The repeated measures ANOVA was based on the log transformed startle eye-blink potentiations (in millivolts) for AN REC in the BAL (Neutral: 227.43 ± 124.19; Underweight: 204.39 ± 105.90; Healthy: 210.52 ± 94.51) and APTD conditions (Neutral: 224.77 ± 113.62; Underweight: 230.48 ± 118.95; Healthy: 223.19 ± 125.10), and for HC in the BAL (Neutral: 229.66 ± 165.67; Underweight: 254.88 ± 168.49; Healthy: 236.91 ± 159.35) and APTD conditions (Neutral: 259.87 ± 131.95; Underweight: 255.34 ± 133.09; Healthy: 255.01 ± 140.09). Data are expressed as Means ± SD. *P ≤ 0.*05*. ANOVA: analysis of variance. SD: standard deviation.

Raw startle amplitudes were log-transformed to meet the assumption of normality. Repeated measures ANOVA with group as the between-subjects factor, and drink and underweight body relative to neutral pictures as the within-subjects factors showed a significant three-way interaction (F(1, 27) = 11.19, p < 0.01, *η*^2^ = 0.29). Similarly, a significant three-way interaction was found for underweight relative to healthy body pictures (F(1, 27) = 4.42, p < 0.05, *η*^2^ = 0.14). Bonferroni corrected and bootstrap methods for *post-hoc* t-tests indicated that, in the BAL AA state, the AN REC showed decreased startle potentiation (an appetitive response) to underweight relative to neutral cues (t (14) = 2.59, p = 0.04, ES = 1.00). The HC had the opposite response (t (13) = -3.14, p = 0.02, ES = 1.21), *i*.*e*. an aversive response. When DA was depleted (APTD), these effects were no longer present. In fact, visual inspection of startle patterns shows that underweight stimuli (relative to neutral cues) were perceived as more aversive in AN REC, while neutral stimuli (relative to underweight cues) were perceived as more aversive in HC; although these differences did not reach significance (Fig 1).

**Fig 2:**
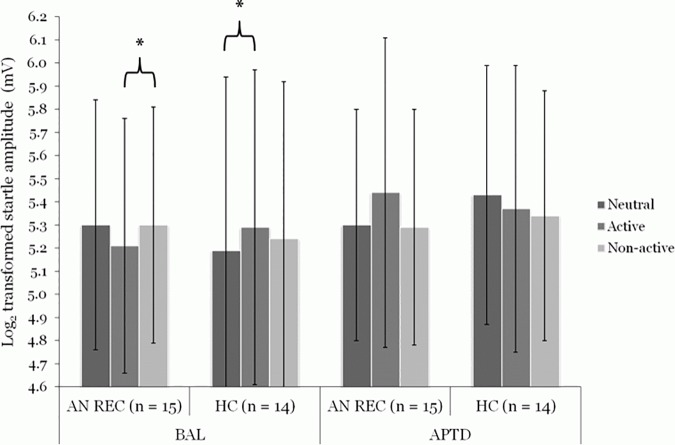
Effect of acute phenylalanine and tyrosine depletion (APTD) on startle eye-blink amplitudes to physically active and non-active body stimuli (relative to neutral cues) in individuals recovered from anorexia nervosa (AN REC, n = 15) and healthy controls (HC, n = 14). In the balanced condition (BAL), AN REC tended towards decreased startle potentiation (an appetitive response) to active (5.21 ± 0.55) relative to non-active cues (5.30 ± 0.51), while HC displayed increased startle potentiation (an aversive response) to active (5.29 ± 0.68) relative to neutral (5.19 ± 0.75) cues. In the low DA condition (APTD), AN REC perceived active stimuli (5.44 ± 0.67) as more aversive than neutral cues (5.30 ± 0.50), while HC perceived neutral stimuli (5.43 ± 0.56) as more aversive than non-active cues (5.34 ± 0.54); however, differences did not reach significance during APTD. The repeated measures ANOVA was based on the log transformed startle eye-blink potentiations (in millivolts) for AN REC in the BAL (Neutral: 227.43 ± 124.19; Active: 207.19 ± 99.74; Non-active: 222.70 ± 101.74) and APTD conditions (Neutral: 224.77 ± 113.62; Active: 292.60 ± 263.04; Non-active: 221.80 ± 113.70) and for HC in the BAL (Neutral: 229.66 ± 165.67; Active: 243.64 ± 162.38; Non-active: 231.55 ± 160.60) and APTD conditions (Neutral: 259.87 ± 131.95; Active: 246.08 ± 113.52; Non-active: 235.52 ± 122.55). Data are expressed as Means ± SD. *P ≤ 0.10. ANOVA: analysis of variance. SD: standard deviation.

Repeated measures ANOVA with group as the between-subjects factor, and drink and active relative to neutral pictures as the within-subjects factors showed a significant three-way interaction (F(1, 27) = 9.68, p < 0.01, *η*^2^ = 0.26). A three-way interaction was also found for active relative to non-active pictures (F(1, 27) = 4.44, p < 0.05, *η*^2^ = 0.14). Bonferroni corrected and bootstrap methods for *post-hoc* t-tests indicated that the AN REC group tended to display a decreased (appetitive) startle response to active relative to non-active cues during BAL (t (14) = -2.23, p = 0.08, ES = 0.86), while HC tended to show increased startle amplitudes (an aversive response) to active relative to neutral stimuli (t (13) = -2.38, p = 0.06, ES = 0.92). In the low DA condition, these effects were no longer observed. Again, visual inspection of the startle patterns shows that active stimuli (relative to non-active cues) were perceived as more aversive in AN REC, while neutral stimuli (relative to active cues) were perceived as more aversive in HC; however, differences were not significant (Fig 2).

### Subjective Ratings for ED-Compatible Stimuli

Subjective picture ratings were consistent with startle responses in the balanced condition. AN REC (relative to HC) rated underweight bodies as more attractive and endorsed the positive value of having an underweight body more strongly. The AN REC group also showed greater appearance and interest ratings for active stimuli compared to HC. Similar ratings were observed in the low DA state (S2 Table).

### Correlation Analyses for Startle Data

Correlational analyses were conducted across both groups (S3 Table). In both AA conditions, relevant correlations were found between startle responses to AN-compatible cues and eating pathology, negative affect, and scores endorsing weight control, attractiveness, tone, and health as important reasons for exercise (S1 Results).

## Discussion

In AN REC and HC, startle eye-blink modulation (SEM) and a DA lowering procedure (ATPD) were used to assess the potential involvement of dopaminergic systems in motivational responses to AN-compatible cues. In a BAL state, the AN REC group showed an appetitive response (lower startle potentiation) to underweight and physically active cues (relative to neutral and non-active cues, respectively). In contrast, in the BAL state, HC had a decreased appetitive response (increased startle potentiation) to underweight and active images relative to neutral stimuli. Importantly, the significant differences observed in the balanced state were no longer present when DA was lowered by ATPD. In fact, in the low DA condition, visual inspection of startle patterns showed that AN REC participants perceived underweight and active stimuli as more aversive (than neutral and non-active cues, respectively), while HC perceived neutral stimuli as more aversive than underweight and active cues. However, differences did not attain statistical significance.

APTD has been reported to decrease reward-associated striatal DA release, an effect proposed to diminish the ability to sustain motivation to obtain reward (*i*.*e*., ‘wanting’) [51]. Indeed, APTD reduces reward sensitivity [52] and motivational approach towards pharmacological [50, 53] and non-pharmacological rewards [49]. Importantly, reduced ‘wanting’ (as produced by APTD) appears to occur in the absence of changes in hedonic reactivity or ‘liking’ [24, 50, 54, 55], suggesting that striatal dopaminergic transmission influences incentive salience independent of conscious processing or pleasure. This is consistent with our observation that the AN REC group (relative to HC) rated underweight and exercise stimuli as more appealing in both the BAL and low DA conditions. Data also support a role for DA in signalling the presence of salient but non-rewarding experiences, including aversive and alerting motivational events [25, 56–58]. Distinct striatal networks seem to have dissociable motivational signals that support encoding of the motivational value and the motivational salience attributed to appetitive and aversive cues [56, 57]. In this scenario, it can be hypothesised that in people with AN, a state develops in which they activate overlapping dopaminergic motivational circuits differently from HC, which may then lead to the attribution of appetitive value to otherwise "normally" aversive stimuli due to differences in long-term cognitive goals (*e*.*g*., the pursuit of thinness and weight-loss). Overall, findings on the behavioural effects of APTD, and on the role of DA in the signalling of salient events, are consistent with our observation that the AN REC participants perceived underweight and active stimuli as more rewarding (relative to HC, who perceived these stimuli as more aversive) during the balanced control condition only; *i*.*e*., differential evaluation of illness-compatible stimuli was no longer apparent when DA was depleted.

Finally, striatal DA neurons are thought to signal unexpected reward stimuli [59], producing motivational approach, and, over time, this facilitates reinforcement learning [60]. In this way, repeated reward-based behaviours (triggered by environmental reward cues) may become entrenched and resistant to change due to interactions between striatal DA and habit-associated parts of the brain (*e*.*g*., corticostriatal circuits) [9–11, 61, 62]. In accord with this hypothesis, a recent study showed that decreasing DA (via APTD) did not reduce motivation to exercise in AN REC, but did so in HC. This suggests that drive to exercise develops into a behaviour that is independent of DA-mediated reward processes and may become dependent on corticostriatal neurocircuitry that regulates automated, habit-like behaviours [63]. Therefore, in the present study, diminished reflexive startle response to underweight and exercise cues in AN REC during BAL (but not during APTD) suggests that, unlike HC, these individuals reflexively appraise such cues as more rewarding and that this is partly DA-dependent. This heightened motivational salience attributed towards AN-compatible cues may result in a pathological drive to engage in illness-related rewarding behaviours that, with time, maintains and perpetuates the disorder.

The present data are consistent with the proposal that part of the problem in AN is one of aberrant reward-based learning, and thus treatment should be directed towards enabling new learning to occur. The present study also gives rise to another important issue, namely that the “pathological” group (AN REC) were recovered from illness. As such, it suggests that the persisting sensitivity to AN-related cues means they remain vulnerable to illness and that alterations in reward processing of disorder-compatible information are trait markers of disease; although, scarring effects of illness cannot be excluded. It also raises the issue of what behavioural/neural changes have occurred in these individuals that have allowed them to recover. Changes in cognitive processing is the most obvious candidate but a more robust explanation will require comparative studies between ill and recovered individuals, both behaviourally and also involving the imaging of neural circuits in the ill and healthy states.

### Strengths and Limitations

This is the first study to examine the effects of lowering dopamine on the objective motivation towards AN-compatible cues in individuals recovered from AN. Studying an AN REC group is of value as data are not confounded by starvation and the APTD method is not easy to conduct in patient populations. Nevertheless, it remains difficult to determine whether the changes observed in this study are a manifestation of a reward-associated trait that contributes to the onset of AN or a “scar” that is the consequence of previous malnutrition and weight loss. Longitudinal studies recruiting individuals prior to illness, as well as during the acute stage, are required to resolve this issue further. The sample size was small, but similar sized groups are sufficient to see APTD effects on behavioural responses to reward [49, 50]. Women recovered from AN who were on a stable dose of SSRI medication were also included, potentially impacting on dopaminergic effects. Although no differences in startle eyeblink potentiations were observed between AN REC who were taking antidepressants and those who were not, the sample size was small and thus it may not have been possible to detect whether antidepressants confounded the results. Standard affective picture stimuli were not included in this task, limiting our ability to compare startle amplitudes for AN-specific stimuli with those established for standardized valence stimuli. In AN REC, cues of underweight bodies and exercise are likely perceived as more appetitive than those of other body types and inactivity for several reasons. For example, images depicting healthy weight may be perceived as less rewarding as they are not consistent with illness-related goals (*i*.*e*., further weight loss). Furthermore, cognitive dissonance may develop during exposure to cues of inactivity in individuals who are physically restless, a state often observed in people with AN who are hyperactive [31, 64]. Therefore, it will be of value for future SEM studies to consider picture selection more carefully (*e*.*g*., adjust stimuli to individual symptomatology to introduce a stronger aspect of self-reference). Moreover, consistent with previous studies, large inter-individual variability in startle eyeblink magnitudes were observed. Considering the marked variability in the measure combined with the relatively small sample size, the risk of type 2 error cannot be omitted. Finally, APTD might affect catecholamine metabolism other than DA (i.*e*., norepinephrine) [65]; however, microdialysis [66, 67], neuroendocrine [68–70], and fos immunocytochemical studies [71] indicate that it preferentially affects DA transmission.

## Conclusions

This study examined the effects of lowering DA on implicit motivation towards images of female bodies and exercise in an AN REC group and HC. Appetitive motivation towards illness-compatible cues was assessed using a startle reflex paradigm. In a BAL amino acid state, AN REC show implicit appetitive motivation for images depicting underweight female bodies and exercise relative to a group of HC. These effects were not observed in a low DA condition (APTD), suggesting the positive value attributed to these cues is influenced by dopaminergic reward processes. This is consistent with reports that illness-compatible cues are perceived as rewarding in AN and adds to the evidence supporting a role for dopaminergic reward/motivation systems in the development and maintenance of such biases in AN.

## Supporting Information

S1 Appendix Mean weights of amino acids for the acute phenylalanine / tyrosine depletion (APTD) method. (DOCX)

S1 Results Pearson correlation analyses for startle data. (DOC)

S1 Table Log transformed startle eye-blink amplitudes to anorexia nervosa (AN)-compatible cues (relative to neutral cues) in individuals recovered from anorexia nervosa who were taking SSRI medication (AN REC SSRI, n = 7), not taking SSRI medication (AN REC, n = 8), and healthy controls (HC, n = 14). (DOCX)

S2 Table Subjective ratings for AN-compatible stimuli. (DOCX)

S3 Table Pearson correlation analyses for startle eye-blink data. (DOCX)
