# Supplementary material for: Evidence that Illness-Compatible Cues Are Rewarding in Women Recovered from Anorexia Nervosa: A Study of the Effects of Dopamine Depletion on Eye-Blink Startle Responses
Source: PLoS One. 2016 Oct 20;11(10):e0165104. doi: 10.1371/journal.pone.0165104 (PMC5072564; doi:10.1371/journal.pone.0165104)
Supplement: S1 Appendix — (DOCX) [file pone.0165104.s001.docx]

**S1 Appendix. Mean weights of amino acids for the acute phenylalanine / tyrosine depletion (APTD) method**

|  |  |  |  |  |  |
| --- | --- | --- | --- | --- | --- |
|  |  |  | APTD |  | placebo |
|  |  |  |  |  |  |
| L-tryptophan | |  | **1.96** |  | **1.96** |
| L-tyrosine | |  | **X** |  | **5.87** |
| L-phenylalanine | |  | **X** |  | **4.85** |
| L-isoleucine | |  | **6.80** |  | **6.80** |
| L-leucine | |  | **11.48** |  | **11.48** |
| L-valine |  |  | **7.57** |  | **7.57** |
| L-alanine | |  | **4.68** |  | **4.68** |
| glycine |  |  | **2.72** |  | **2.72** |
| L-histidine | |  | **2.72** |  | **2.72** |
| L-Lysine monohydrochloride | | | **9.35** |  | **9.35** |
| L-proline | |  | **10.37** |  | **10.37** |
| L-serine |  |  | **5.87** |  | **5.87** |
| L-threonine | |  | **5.53** |  | **5.53** |
| L-arginine | |  | **4.17** |  | **4.17** |
| L-cysteine | |  | **2.30** |  | **2.30** |
| L-methionine | |  | **2.55** |  | **2.55** |
|  |  |  |  |  |  |
| Total active mixture | | | **72.51** |  | **83.22** |
