# Supplementary material for: Evidence that Illness-Compatible Cues Are Rewarding in Women Recovered from Anorexia Nervosa: A Study of the Effects of Dopamine Depletion on Eye-Blink Startle Responses
Source: PLoS One. 2016 Oct 20;11(10):e0165104. doi: 10.1371/journal.pone.0165104 (PMC5072564; doi:10.1371/journal.pone.0165104)
Supplement: S1 Results — (DOC) [file pone.0165104.s002.doc]

## S1 Results. Pearson correlation analyses for startle data

Correlational analyses were conducted across both groups (Table in S2 Table). In the balanced condition, there were negative relationships between underweight body cue startle difference scores (*i.e.,* subtracting startle amplitudes for neutral cues from those for underweight body cues) and EDE-Q global scores (r = -0.82, p < 0.01), DASS-total scores (r = -0.61, p = 0.03), and scores endorsing weight control (r = -0.68, p = 0.01), attractiveness (r = -0.56, p < 0.05), and tone (r = -0.69, p = 0.01) as important reasons for exercise. This indicates that participants reporting higher ED pathology, mood pathology, and scores related to appearance/weight management as important reasons for exercise, showed lower startle potentiation (an appetitive response) to underweight relative to neutral stimuli. During APTD however, underweight body cue difference scores showed negative correlations with BMI (r = -0.64, p = 0.02), suggesting that higher weight was associated with lower startle (an appetitive response) to underweight (relative to neutral) cues in a low DA state.

During BAL, negative relationships were also observed between healthy body cue startle difference scores (*i.e.,* subtracting startle amplitudes for neutral cues from those for healthy body cues) and EDE-Q global scores (r = -0.56, p < 0.05) and ratings endorsing weight control (r = -0.57, p = 0.04), attractiveness (r = -0.74, p < 0.01), and tone (r = -0.57, p = 0.04) as important reasons for exercise. This suggests that individuals reporting higher ED pathology and scores related to appearance/weight control as important reasons for exercise also showed decreased startle amplitudes (an appetitive response) to healthy bodies relative to neutral cues. During APTD, difference scores for healthy bodies correlated negatively with BMI (r = -0.57, p = 0.04) and positively with scores endorsing improving mood (r = 0.58, p = 0.04) as a reason for exercise, indicating that, in a low DA state, higher weight was associated with lower startle potentiation (an appetitive response) to healthy (relative to neutral) cues, while exercising as a means to improve mood was associated with increased startle (an aversive response) to these cues.

Finally, during BAL, there were negative relationships between active cue startle difference scores (*i.e.,* subtracting startle amplitudes for neutral cues from those for physically active stimuli) and EDE-Q global (r = -0.69, p = 0.01) and DASS-total (r = -0.55, p = 0.05) scores, indicating that higher eating and mood pathology were associated with lower startle potentiation (an appetitive response) to active versus neutral stimuli. During BAL, there was also a positive relationship between these scores and ratings endorsing improving health (r = 0.55, p = 0.05) as a reason for exercise, suggesting that exercising more for health reasons was associated with a higher, more aversive startle response to active (relative to neutral) cues. In contrast, during APTD, active cue difference scores correlated negatively with BMI (r = -0.77, p < 0.01) and ratings for improving health (r = -0.56, p < 0.05) as a reason for exercise, indicating that higher weight and exercising as a means to improve health were associated with lower startle potentiation (an appetitive response) to active (relative to neutral) cues in a low DA state.
