# Supplementary material for: Evidence that Illness-Compatible Cues Are Rewarding in Women Recovered from Anorexia Nervosa: A Study of the Effects of Dopamine Depletion on Eye-Blink Startle Responses
Source: PLoS One. 2016 Oct 20;11(10):e0165104. doi: 10.1371/journal.pone.0165104 (PMC5072564; doi:10.1371/journal.pone.0165104)
Supplement: S1 Table — (DOCX) [file pone.0165104.s003.docx]

**S1 Table. Log transformed startle eye-blink amplitudes to anorexia nervosa (AN)-compatible cues (and neutral cues) in individuals recovered from anorexia nervosa who were taking SSRI medication (AN REC SSRI, n = 9), not taking SSRI medication (AN REC, n = 8), and healthy controls (HC, n = 14) in the balanced (BAL) and acute phenylalanine / tyrosine depletion (APTD) states**

| **Startle Amplitude (in A/D units)** | | **AN REC SSRI**  **(n = 7)** | **AN REC**  **(n = 8)** | **HC**  **(n = 614** | **ANOVA** |
| --- | --- | --- | --- | --- | --- |
| Neutral | BAL | 5.43 ± 0.63 | 5.17 ± 0.46 | 5.19 ± 0.75 | F(2) = 0.38, p = 0.69 |
|  | APTD | 5.38 ± 0.51 | 5.24 ± 0.52 | 5.43 ± 0.56 | F(2) = 0.45, p = 0.64 |
| Underweight | BAL | 5.29 ± 0.70 | 5.09 ± 0.49 | 5.35 ± 0.65 | F(2) = 0.06, p = 0.94 |
|  | APTD | 5.34 ± 0.57 | 5.29 ± 0.54 | 5.38 ± 0.69 | F(2) = 0.19, p = 0.83 |
| Healthy | BAL | 5.29 ± 0.64 | 5.20 ± 0.42 | 5.29 ± 0.63 | F(2) = 0.16, p = 0.85 |
|  | APTD | 5.33 ± 0.59 | 5.20 ± 0.61 | 5.37 ± 0.70 | F(2) = 0.34, p = 0.71 |
| Active | BAL | 5.29 ± 0.66 | 5.13 ± 0.47 | 5.29 ± 0.68 | F(2) = 0.05, p = 0.96 |
|  | APTD | 5.38 ± 0.54 | 5.49 ± 0.81 | 5.37 ± 0.62 | F(2) = 0.18, p = 0.83 |
| Non-Active | BAL | 5.38 ± 0.55 | 5.23 ± 0.49 | 5.24 ± 0.68 | F(2) = 0.08, p = 0.92 |
|  | APTD | 5.41 ± 0.49 | 5.18 ± 0.53 | 5.34 ± 0.54 | F(2) = 0.41, p = 0.67 |
