# Supplementary material for: Evidence that Illness-Compatible Cues Are Rewarding in Women Recovered from Anorexia Nervosa: A Study of the Effects of Dopamine Depletion on Eye-Blink Startle Responses
Source: PLoS One. 2016 Oct 20;11(10):e0165104. doi: 10.1371/journal.pone.0165104 (PMC5072564; doi:10.1371/journal.pone.0165104)
Supplement: S2 Table — (DOCX) [file pone.0165104.s004.docx]

## S2 Table. Subjective ratings for AN-compatible stimuli

| **VAS Rating** | | **AN REC (n = 17)** | **HC (n = 15)** | **ANOVA ME Group** | **ANOVA ME Picture** | **ANOVA ME Drink** | **ANOVA Group x Picture** | **ANOVA Group x Drink** | **ANOVA Picture x Drink** | **ANOVA Group x Picture x Drink** |
| --- | --- | --- | --- | --- | --- | --- | --- | --- | --- | --- |
| Underweight vs. Neutral Appearance | U BAL N BAL U APTD N APTD | 3.63 ± 2.29 4.73 ± 1.92 3.98 ± 2.50 4.77 ± 1.55 | 1.85 ± 1.61 5.01 ± 2.41 1.89 ± 1.85 4.79 ± 2.68 | F(1) = 2.79 p = 0.11 η^2^ = 0.09 | ****** F(1) = 15.50 p < 0.01 η^2^ = 0.35 | F(1) = 0.08 p = 0.78 η^2^ < 0.01 | ***** F(1) = 4.27 p = 0.05 η^2^ = 0.13 | F(1) = 0.60 p = 0.45 η^2^ = 0.02 | F(1) = 0.97 p = 0.33 η^2^ = 0.03 | F(1) < 0.01 p = 0.94 η^2^ < 0.01 |
| Healthy vs. Neutral Appearance | H BAL N BAL H APTD N APTD | 6.89 ± 1.52 4.73 ± 1.92 6.89 ± 1.32 4.77 ± 1.55 | 6.56 ± 1.25 5.01 ± 2.41 6.66 ± 1.57 4.79 ± 2.68 | F(1) = 0.03 p = 0.87 η^2^ < 0.01 | ****** F(1) = 18.04 p < 0.01 η^2^ = 0.38 | F(1) = 0.01 p = 0.91 η^2^ < 0.01 | F(1) = 0.22 p = 0.64 η^2^ < 0.01 | F(1) = 0.05 p = 0.83 η^2^ < 0.01 | F(1) = 0.16 p = 0.69 η^2^ = 0.01 | F(1) = 0.24 p = 0.63 η^2^ = 0.01 |
| Active vs. Neutral Appearance | A BAL N BAL A APTD N APTD | 5.11 ± 2.14 4.73 ± 1.92 5.23 ± 1.91 4.77 ± 1.55 | 4.54 ± 2.24 5.01 ± 2.41 4.62 ± 2.54 4.79 ± 2.68 | F(1) = 0.14 p = 0.71 η^2^ < 0.01 | F(1) = 0.02 p = 0.90 η^2^ < 0.01 | F(1) < 0.01 p = 0.99 η^2^ < 0.01 | F(1) = 0.72 p = 0.40 η^2^ = 0.03 | F(1) = 0.13 p = 0.72 η^2^ < 0.01 | F(1) = 0.24 p = 0.63 η^2^ < 0.01 | F(1) = 0.07 p = 0.79 η^2^ < 0.01 |
| Active vs. Neutral Interest | A BAL N BAL A APTD N APTD | 5.06 ± 2.38 4.73 ± 1.92 5.05 ± 2.22 4.77 ± 1.55 | 4.31 ± 2.41 5.01 ± 2.41 4.38 ± 2.88 4.79 ± 2.68 | F(1) = 0.08 p = 0.79 η^2^ < 0.01 | F(1) = 1.40 p = 0.25 η^2^ = 0.05 | F(1) = 0.23 p = 0.64 η^2^ < 0.01 | F(1) = 1.37 p = 0.25 η^2^ = 0.05 | F(1) = 0.05 p = 0.82 η^2^ < 0.01 | F(1) = 0.13 p = 0.72 η^2^ < 0.01 | F(1) < 0.01 p = 0.97 η^2^ < 0.01 |
| Active vs. Neutral Pleasure | A BAL N BAL A APTD N APTD | 4.89 ± 2.22 4.73 ± 1.92 5.27 ± 2.00 4.77 ± 1.55 | 4.51 ± 2.37 5.01 ± 2.41 4.56 ± 2.80 4.79 ± 2.68 | F(1) = 0.11 p = 0.75 η^2^ < 0.01 | F(1) = 0.72 p = 0.40 η^2^ = 0.02 | F(1) = 0.51 p = 0.48 η^2^ = 0.02 | F(1) = 0.52 p = 0.48 η^2^ < 0.02 | F(1) = 0.62 p = 0.44 η^2^ = 0.02 | F(1) = 0.09 p = 0.77 η^2^ < 0.01 | F(1) < 0.01 p = 0.99 η^2^ < 0.01 |
| Non-Active vs. Neutral Appearance | NA BAL N BAL NA APTD N APTD | 2.64 ± 1.47 4.73 ± 1.92 2.38 ± 1.33 4.77 ± 1.55 | 3.59 ± 2.26 5.01 ± 2.41 3.05 ± 2.36 4.79 ± 2.68 | F(1) = 0.79 p = 0.38 η^2^ = 0.03 | ****** F(1) = 26.09 p < 0.01 η^2^ = 0.47 | F(1) = 1.12 p = 0.30 η^2^ = 0.04 | F(1) = 0.77 p = 0.39 η^2^ = 0.03 | F(1) = 0.33 p = 0.57 η^2^ = 0.01 | F(1) = 0.50 p = 0.49 η^2^ = 0.02 | F(1) < 0.01 p = 0.98 η^2^ < 0.01 |
| Non-Active vs. Neutral Interest | NA BAL N BAL NA APTD N APTD | 2.24 ± 1.51 4.73 ± 1.92 2.05 ± 1.19 4.77 ± 1.55 | 2.93 ± 2.07 5.01 ± 2.41 2.51 ± 2.12 4.79 ± 2.68 | F(1) = 0.71 p = 0.41 η^2^ = 0.02 | ** F(1) = 21.48 p < 0.01 η^2^ = 0.43 | F(1) = 0.07 p = 0.79 η^2^ < 0.01 | F(1) = 0.10 p = 0.76 η^2^ < 0.01 | F(1) = 0.02 p = 0.90 η^2^ < 0.01 | F(1) = 1.16 p = 0.29 η^2^ = 0.04 | F(1) = 0.16 p = 0.69 η^2^ < 0.01 |
| Non-Active vs. Neutral Pleasure | NA BAL N BAL NA APTD N APTD | 2.66 ± 1.58 4.73 ± 1.92 2.42 ± 1.42 4.77 ± 1.55 | 3.48 ± 2.37 5.01 ± 2.41 2.95 ± 2.40 4.79 ± 2.68 | F(1) = 0.48 p = 0.50 η^2^ = 0.02 | ****** F(1) = 13.93 p < 0.01 η^2^ = 0.33 | F(1) = 0.42 p = 0.52 η^2^ = 0.01 | F(1) = 0.44 p = 0.52 η^2^ = 0.02 | F(1) = 0.44 p = 0.51 η^2^ = 0.02 | F(1) = 1.13 p = 0.30 η^2^ = 0.04 | F(1) < 0.01 p = 0.99 η^2^ < 0.01 |
| Underweight vs. Healthy Attractive | U BAL H BAL U APTD H APTD | 3.63 ± 2.29 6.89 ± 1.52 3.98 ± 2.50 6.89 ± 1.32 | 1.85 ± 1.61 6.56 ± 1.25 1.89 ± 1.85 6.66 ± 1.57 | ***** F(1) = 6.28 p = 0.02 η^2^ = 0.18 | ** F(1) = 82.66 p < 0.01 η^2^ = 0.74 | F(1) = 0.67 p = 0.42 η^2^ = 0.02 | F(1) = 3.70 p = 0.06 η^2^ = 0.11 | F(1) = 0.11 p = 0.74 η^2^ < 0.01 | F(1) = 0.22 p = 0.64 η^2^ < 0.01 | F(1) = 0.44 p = 0.51 η^2^ = 0.02 |
| Underweight vs. Healthy Body Size | U BAL H BAL U APTD H APTD | 1.51 ± 0.43 2.69 ± 0.94 1.75 ± 0.79 2.65 ± 1.05 | 1.44 ± 0.35 2.68 ± 0.61 1.39 ± 0.37 2.61 ± 0.55 | F(1) = 0.29 p = 0.60 η^2^ = 0.01 | ** F(1) = 162.16 p < 0.01 η^2^ = 0.85 | F(1) = 0.14 p = 0.71 η^2^ < 0.01 | F(1) = 1.14 p = 0.30 η^2^ = 0.04 | F(1) = 1.83 p = 0.19 η^2^ = 0.06 | F(1) = 2.29 p = 0.14 η^2^ = 0.07 | F(1) = 1.82 p = 0.19 η^2^ = 0.06 |
| Underweight vs. Healthy Body Size | U BAL H BAL U APTD H APTD | 4.39 ± 2.79 7.20 ± 1.33 4.81 ± 2.71 6.94 ± 1.39 | 2.22 ± 1.97 6.56 ± 1.34 2.01 ± 1.99 6.85 ± 1.52 | ** F(1) = 8.40 p < 0.01 η^2^ = 0.23 | ** F(1) = 59.11 p < 0.01 η^2^ = 0.67 | F(1) = 0.11 p = 0.75 η^2^ < 0.01 | * F(1) = 5.31 p = 0.03 η^2^ = 0.16 | F(1) = 0.01 p = 0.91 η^2^ < 0.01 | F(1) = 0.08 p = 0.78 η^2^ < 0.01 | F(1) = 3.27 p = 0.08 η^2^ = 0.10 |
| Active vs. Non-Active Appearance | A BAL NA BAL A APTD NA APTD | 5.11 ± 2.14 2.64 ± 1.47 5.23 ± 1.91 2.38 ± 1.33 | 4.54 ± 2.24 3.59 ± 2.26 4.62 ± 2.54 3.05 ± 2.36 | F(1) = 0.04 p = 0.84 η^2^ < 0.01 | ** F(1) = 26.58 p < 0.01 η^2^ = 0.48 | F(1) = 0.31 p = 0.59 η^2^ = 0.01 | F(1) = 3.36 p = 0.08 η^2^ = 0.10 | F(1) = 0.09 p = 0.77 η^2^ < 0.01 | F(1) = 1.84 p = 0.19 η^2^ = 0.06 | F(1) = 0.10 p = 0.76 η^2^ < 0.01 |
| Active vs. Non-Active Interest | A BAL NA BAL A APTD NA APTD | 5.06 ± 2.38 2.24 ± 1.51 5.05 ± 2.22 2.05 ± 1.19 | 4.31 ± 2.41 2.93 ± 2.07 4.38 ± 2.88 2.51 ± 2.12 | F(1) = 0.01 p = 0.91 η^2^ < 0.01 | ** F(1) = 39.55 p < 0.01 η^2^ = 0.58 | F(1) = 0.30 p = 0.59 η^2^ = 0.01 | F(1) = 3.15 p = 0.08 η^2^ = 0.10 | F(1) = 0.02 p = 0.88 η^2^ < 0.01 | F(1) = 0.54 p = 0.47 η^2^ = 0.02 | F(1) = 0.12 p = 0.73 η^2^ < 0.01 |
| Active vs. Non-Active Pleasure | A BAL NA BAL A APTD NA APTD | 4.89 ± 2.22 2.66 ± 1.58 5.27 ± 2.00 2.42 ± 1.42 | 4.51 ± 2.37 3.48 ± 2.37 4.56 ± 2.80 2.95 ± 2.40 | F(1) = 0.01 p = 0.91 η^2^ < 0.01 | ** F(1) = 22.78 p < 0.01 η^2^ = 0.44 | F(1) = 0.11 p = 0.75 η^2^ < 0.01 | F(1) = 2.27 p = 0.14 η^2^ = 0.07 | F(1) = 0.34 p = 0.57 η^2^ = 0.01 | F(1) = 2.15 p = 0.15 η^2^ = 0.07 | F(1) < 0.01 p = 0.97 η^2^ < 0.01 |

*Legend:* 10 cm VAS ratings in response to neutral, underweight female body, healthy female body, active, and non-active pictorial stimuli in AN REC and HC groups. Results are reported for both the BAL and the APTD amino acid drink conditions. Data are expressed as Means ± SD. *** P ≤ 0.01, * P ≤ 0.05.* A: active stimuli. ANOVA: analysis of variance. AN REC: anorexia nervosa recovered. APTD: acute phenylalanine/tyrosine depletion condition. BAL: balanced amino acid condition. H: healthy weight stimuli. HC: healthy controls. ME: main effect. N: neutral stimuli. NA: non-active stimuli. SD= standard deviation. U: underweight stimuli. VAS: visual analogue scales. X: interaction effect.
