# Supplementary material for: Evidence that Illness-Compatible Cues Are Rewarding in Women Recovered from Anorexia Nervosa: A Study of the Effects of Dopamine Depletion on Eye-Blink Startle Responses
Source: PLoS One. 2016 Oct 20;11(10):e0165104. doi: 10.1371/journal.pone.0165104 (PMC5072564; doi:10.1371/journal.pone.0165104)
Supplement: S3 Table — (DOCX) [file pone.0165104.s005.docx]

**S3 Table. Pearson correlation analyses for startle eye-blink data**

| Picture stimuli | | BMI | EDE-Q Global | DASS-Total | REI Weight Control | REI Attractive | REI Tone | REI Health | REI Fitness | REI Mood | REI Enjoyment | Exercise hrs/wk |
| --- | --- | --- | --- | --- | --- | --- | --- | --- | --- | --- | --- | --- |
| Underweight body | BAL APTD | r = 0.04 p = 0.90 | ** r = -0.82 p < 0.01 | * r = -0.61 p = 0.03 | ** r = -0.68 p = 0.01 | * r = -0.56 p < 0.05 | ** r = -0.69 p = 0.01 | r = 0.48 p = 0.10 | r = -0.03 p = 0.93 | r = -0.49 p = 0.13 | r = 0.30 p = 0.32 | r = -0.29 p = 0.33 |
|  |  | * r = -0.64 p = 0.02 | r = 0.25 p = 0.42 | r = 0.36 p = 0.23 | r = 0.49 p = 0.09 | r = 0.29 p = 0.34 | r = 0.43 p = 0.14 | r = -0.39 p = 0.18 | r = -0.06 p = 0.86 | r = 0.50 p = 0.09 | r = -0.12 p = 0.70 | r = -0.24 p = 0.42 |
| Healthy body | BAL APTD | r = -0.36 p = 0.22 | * r = -0.56 p < 0.05 | r = -0.42 p = 0.15 | * r = -0.57 p = 0.04 | ** r = -0.74 p < 0.01 | * r = -0.57 p = 0.04 | r = 0.10 p = 0.74 | r = 0.06 p = 0.84 | r = -0.30 p = 0.31 | r = 0.25 p = 0.42 | r = -0.17 p = 0.58 |
|  |  | * r = -0.57 p = 0.04 | r = 0.20 p = 0.51 | r = 0.26 p = 0.39 | r = 0.38 p = 0.20 | r = 0.18 p = 0.55 | r = 0.32 p = 0.28 | r = -0.29 p = 0.34 | r = -0.22 p = 0.47 | * r = 0.58 p = 0.04 | r = -0.09 p = 0.78 | r = -0.18 p = 0.55 |
| Active body | BAL APTD | r = 0.13 p = 0.68 | ** r = -0.69 p = 0.01 | * r = -0.55 p = 0.05 | r = -0.49 p = 0.09 | r = -0.43 p = 0.14 | r = -0.41 p = 0.17 | * r = 0.55 p = 0.05 | r = 0.25 p = 0.42 | * r = -0.33 p = 0.27 | r = 0.36 p = 0.22 | r = -0.22 p = 0.48 |
|  |  | ** r = -0.77 p < 0.01 | r = 0.18 p = 0.56 | r = 0.27 p = 0.38 | r = 0.16 p = 0.60 | r = -0.17 p = 0.59 | r = 0.08 p = 0.80 | * r = -0.56 p < 0.05 | r = -0.13 p = 0.68 | r = 0.30 p = 0.32 | r = -0.01 p = 0.96 | r = -0.18 p = 0.55 |
| Non-active body | BAL APTD | r = -0.03 p = 0.93 | r = -0.07 p = 0.82 | r = 0.05 p = 0.88 | r = -0.30 p = 0.31 | r = -0.51 p = 0.07 | r = -0.30 p = 0.31 | r = 0.16 p = 0.69 | r = 0.05 p = 0.87 | r = 0.08 p = 0.80 | r = 0.19 p = 0.54 | r = 0.18 p = 0.56 |
|  |  | r = -0.33 p = 0.27 | r = 0.26 p = 0.38 | r = 0.13 p = 0.68 | r = 0.22 p = 0.47 | r = -0.03 p = 0.91 | r = 0.30 p = 0.31 | r = -0.45 p = 0.12 | r = 0.10 p = 0.76 | r = 0.41 p = 0.17 | r = 0.03 p = 0.93 | r = 0.14 p = 0.65 |

*Legend:* Pearson correlations between startle difference scores [subtracting startle amplitudes for neutral cues from those for body (underweight, healthy) and exercise (active, non-active) cues] during the BAL and APTD conditions, and BMI, eating pathology as measured by the EDE-Q, mood as measured by the DASS-21, reasons for exercise as measured by the REI, and baseline exercise experience as measured by exercise hours/week. Data are reported for the anorexia nervosa recovered and healthy control groups as a whole. *** P ≤ 0.01, * P ≤ 0.05.* APTD: acute phenylalanine/tyrosine depletion condition. BAL: balanced amino acid condition. BMI: Body Mass Index. DASS: Depression, Anxiety, and Stress Scales. EDE-Q: Eating Disorders Examination Questionnaire. REI: Reasons for Exercise Inventory.
